# Portable Breathing Monitoring With Phase‐Resolved Airflow Dynamics Enabled by a Dual‐Response Flexible PZT Sensor

**DOI:** 10.1002/adhm.71282

**Published:** 2026-05-28

**Authors:** Minyu Li, Jun Aoyama, Yuchao Wu, Tomomi Uchiyama, Kosho Yoshikawa, Toshiki Mano, Yuxi Song, Hedong Zhang

**Affiliations:** ^1^ Department of Complex Systems Science Graduate School of Informatics Nagoya University Nagoya Japan; ^2^ Institute of Materials and Systems for Sustainability Nagoya University Nagoya Japan; ^3^ Department of Respiratory Medicine Daido Hospital Kojunkai Social Medical Corporation Nagoya Japan; ^4^ Chuo Graduate School of Strategic Management Chuo University Tokyo Japan

**Keywords:** flexible PZT sensor, intra‐cycle analysis, phase‐resolved breathing dynamics, piezoelectric–pyroelectric coupling, portable healthcare devices, Respiratory monitoring

## Abstract

Respiratory monitoring in daily‐life settings is important for health assessment, yet extracting physiologically interpretable information from breathing signals under natural conditions remains challenging, as breathing is inherently dynamic and strongly modulated by behavior. Here, a portable breathing monitoring device based on a flexible lead zirconate titanate sensor is developed to address this challenge. By exploiting polarity‐opposed piezoelectric and pyroelectric responses through sensor orientation, the recorded breathing waveform exhibits a characteristic dual‐component structure, consisting of a narrow transient spike followed by a broad quasi‐steady peak within each breathing phase. This intrinsic waveform structure enables phase‐resolved quantification of how breathing effort is distributed between transient and quasi‐steady components during inhalation and exhalation. Pilot measurements and exploratory analyses in healthy subjects and patients with chronic obstructive pulmonary disease or asthma suggest a phase‐specific redistribution of breathing dynamics in patients, mainly characterized by a coupled decrease in quasi‐steady inhalation contribution and an increase in transient exhalation contribution. By transforming complex breathing dynamics into stable and physiologically meaningful signal components, this dual‐response sensing approach enables more robust access to function‐related changes during natural breathing in daily life.

## Graphical abstract

A portable respiratory monitoring device based on a flexible PZT sensor captures pressure‐ and temperature‐related airflow responses in a single waveform. Polarity‐opposed piezoelectric and pyroelectric components generate a narrow transient spike and a broad quasi‐steady peak during both inhalation and exhalation. This dual‐component structure enables intra‐cycle redistribution metrics that quantify relative transient and quasi‐steady breathing contributions.

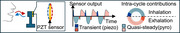

## 1 Introduction

Respiration is one of the most fundamental vital signs and forms a tightly coupled regulatory loop with autonomic and cardiovascular systems [1, 2, 3, 4]. In addition to respiratory diseases such as chronic obstructive pulmonary disease (COPD) and asthma [5, 6], respiratory rate and pattern changes have been shown to be closely associated with cardiovascular function and overall systemic health [1, 2, 3, 7]. Previous studies have indicated that subtle alterations in respiratory rate or detailed breathing patterns may precede detectable changes in conventional clinical indicators such as heart rate and blood pressure, suggesting respiration as a sensitive early marker of health deterioration [2, 8, 9, 10]. Therefore, respiratory monitoring, particularly that can be performed repeatedly and naturally in daily life, is of great importance for long‐term health assessment and more personalized clinical management [1, 11, 12].

At present, spirometry remains the clinical gold standard for evaluating respiratory function. Directly measuring airflow under controlled conditions, it provides quantitative indices such as forced expiratory volume and vital capacity that are well established in clinical practice [5, 6, 13]. However, spirometry relies on forced breathing tasks, strict subject cooperation, and bulky instrumentation, which fundamentally limit its applicability for repeated measurements and assessment under natural daily‐life conditions [1, 11, 12, 14]. As a result, despite the recognized clinical importance of respiration, detailed respiratory information is rarely captured outside clinical or laboratory settings.

To overcome these limitations, a variety of approaches for respiratory monitoring in daily‐life settings have been actively developed in recent years [1, 11, 12, 15, 16]. These approaches can be broadly classified into indirect, non–airflow‐based methods and direct airflow‐sensing methods. Non–airflow‐based approaches infer respiration from body motion, most commonly chest or abdominal movement, and are typically implemented using inertial measurement units or strain‐based wearable sensors [17, 18, 19, 20, 21, 22, 23]. These methods enable continuous and unobtrusive monitoring and have therefore been widely explored for long‐term use. However, because the primary measured signals are mechanical body movements rather than respiratory airflow, non–airflow‐based methods are intrinsically less sensitive to airflow‐related physiological properties such as airway resistance, lung compliance, and flow limitation, which are central to respiratory pathophysiology [1, 5, 6].

Direct airflow sensing, in principle, offers higher physiological relevance and closer correspondence to spirometric measurements [1, 15, 24]. Accordingly, a variety of portable and wearable airflow sensors have been proposed in recent years [25, 26, 27, 28, 29]. To date, most airflow‐based respiratory monitoring studies have focused on steady‐state or quasi‐steady parameters, such as respiratory rate, tidal volume, or peak expiratory flow, which primarily characterize the overall breathing rate and depth [1, 11, 30, 31]. However, airflow during everyday natural (tidal) breathing is strongly modulated by behavior and inherently time‐varying rather than steady. In particular, transitions between inhalation and exhalation involve rapid airflow changes governed by airway mechanics, lung compliance, and neuromuscular control [5, 6, 7, 32, 33, 34, 35, 36]. Emerging evidence indicates that dynamic features associated with inhalation–exhalation phase transitions may offer increased sensitivity to respiratory instability and early deterioration than classical steady‐state metrics such as respiratory rate [37]. Despite their physiological relevance, such transient airflow features have received relatively little attention in daily‐life breathing monitoring. This is partly because conventional sensing approaches are not specifically optimized to reliably capture fast, transient airflow‐related phenomena under unconstrained conditions.

From a physical perspective, breathing airflow inherently involves coupled mechanical and thermal effects, arising from pressure fluctuations and the temperature difference between inhaled and exhaled air. Capturing both aspects, rather than either one alone, is therefore expected to yield richer waveform features that reflect not only steady‐state but also dynamic characteristics of airflow. Motivated by this consideration, we propose a portable breathing monitoring device based on a flexible lead zirconate titanate (PZT) sensor that simultaneously exploits piezoelectric and pyroelectric responses. Rather than allowing the two effects to merge into an indistinguishable superposition, their polarities are intentionally arranged to preserve mutually discernible contributions within a single waveform. As a result, the device produces waveforms that differ qualitatively from those of conventional airflow‐sensing devices, enabling the initial transient and subsequent quasi‐steady phases of each inhalation and exhalation cycle to be distinctly represented under natural oral breathing conditions. By transforming complex, time‐varying airflow dynamics into stable and separable signal components, this device enables robust, phase‐resolved analysis of intra‐cycle breathing behavior for daily‐life monitoring.

## 2 Results and Discussion

### 2.1 Sensor Selection and Structural Design

**FIGURE 1:**
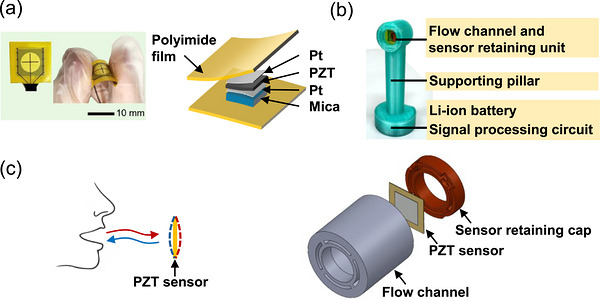
Overview of the proposed portable breathing monitoring device using a flexible PZT sensor. (a) Photographs of the flexible PZT sensor (left) and schematic illustration of its multilayer configuration (right) adapted from Ref. [38] under the terms of the Creative Commons Attribution (CC BY 4.0) license. (b) Photograph of the proposed device. (c) Schematic illustration of the sensor orientation relative to the oral breathing airflow (left) and exploded view of the sensor retaining unit (right).

A flexible PZT sensor (Figure 1a), which exhibits both piezoelectric and pyroelectric responses and was used in our previous study for arterial pulse waveform measurement [38], was employed in this study. In contrast to rigid bulk PZT sensors that primarily operate in the *e*_33_ mode, flexible PZT sensors can undergo pronounced in‐plane deformation (*e*_31_ mode) even under small pressure fluctuations [39], enabling sensitive detection of weak airflow‐induced mechanical stimuli during natural breathing. Compared with widely used organic polyvinylidene fluoride (PVDF) flexible sensors, the flexible PZT sensor used in this study exhibits one‐order‐of‐magnitude higher piezoelectric sensitivity [38] and comparable pyroelectric sensitivity (Note S1). Its long‐term operational stability has also been confirmed in our previous study through repeated bending over 12,000 cycles [38].

As an initial proof‐of‐concept, a portable prototype was developed with a simple structure to realize a well‐defined flow path toward the sensor. Figure 1b shows a photograph of the prototype used for monitoring natural oral breathing in this study. The device consists of a flow channel, a sensor retaining unit, a supporting pillar, and a base housing a Li‐ion battery and a signal processing circuit. As demonstrated in Movie S1 (Supporting Information), an attachable, commercially available mouthpiece for nebulizers is connected to the upstream side of the flow channel to guide airflow toward the sensor during natural oral breathing. The electrical charges generated by the sensor are subsequently processed and transmitted wirelessly via Bluetooth by the circuit in the base to a mobile terminal, such as a tablet or smartphone, for real‐time monitoring and recording.

**FIGURE 2:**
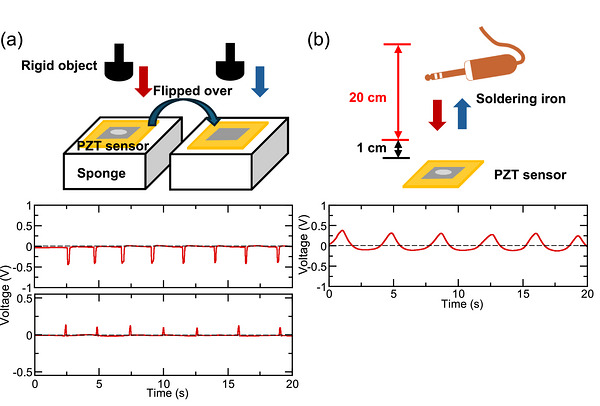
Schematic illustrations of polarity identification experiments under mechanical (a) and thermal (b) stimuli, together with the corresponding voltage outputs. Mechanical stimulus (a): the PZT sensor was placed on a soft sponge and repeatedly pressed using a handheld rounded rigid object, with the sensor tested in both original and inverted orientations to confirm signal polarity reversal. Thermal stimulus (b): a soldering iron was periodically moved vertically above the sensor within a distance range of 1–21 cm over 2 s in each direction, inducing a cyclic temperature variation of the sensor surface between 25.0 and 36.4°C, as measured by an infrared thermometer.

Among these components, the structural design of the sensing unit plays a central role in determining the signal characteristics. To ensure effective mechanical and thermal interaction with the breathing airflow, the sensor was oriented with its surface perpendicular to the airflow direction (Figure 1c, left). Because the sensor has an asymmetric multilayer configuration with a PZT‐on‐mica core (Figure 1a), reversing the sensor orientation changes which side of the core faces the upstream airflow. The orientation was therefore carefully designed so that the piezoelectric and pyroelectric signal components exhibit opposite polarities, preventing signal overlap and enhancing distinguishable features in the measured waveforms. The piezoelectric response is governed by the strain polarity of the PZT layer, with compressive and tensile strains generating signals of opposite polarity. As the mechanical neutral plane of the sensor lies within the mica substrate layer (Note S2), the strain polarity of the PZT layer varies with the sensor orientation relative to the airflow. Specifically, when the PZT layer faces upstream, exhalation generates compressive strain and inhalation generates tensile strain, whereas reversing the sensor orientation inverts the strain polarity and consequently the polarity of piezoelectric signals. In contrast, the pyroelectric response originates from temperature variations during breathing. Exhalation‐induced heating and inhalation‐induced cooling produce signals of opposite polarity; however, this polarity behavior is independent of the sensor orientation. Based on these considerations and the signal polarities independently identified using purely mechanical or thermal stimuli (Figure 2), the sensor was oriented with the PZT layer facing upstream.

The flexible PZT sensor was placed in a sensor seat at the outlet of the flow channel and secured by a retaining cap (Figure 1c, right), which mechanically clamps the sensor along its perimeter and maintains its planar configuration. Four fan‐shaped openings were symmetrically arranged around the sensor to serve as airflow passages. Reducing the opening size would enhance both mechanical and thermal interactions between the sensor and airflow, leading to stronger sensor signals; however, excessively small openings would increase flow resistance and compromise breathing comfort. Therefore, the opening size was designed as the minimum required to maintain smooth and unobstructed natural breathing.

### 2.2 Breathing Waveform Characteristics in Healthy Subjects

#### 2.2.1 Representative Breathing Waveform

**FIGURE 3:**
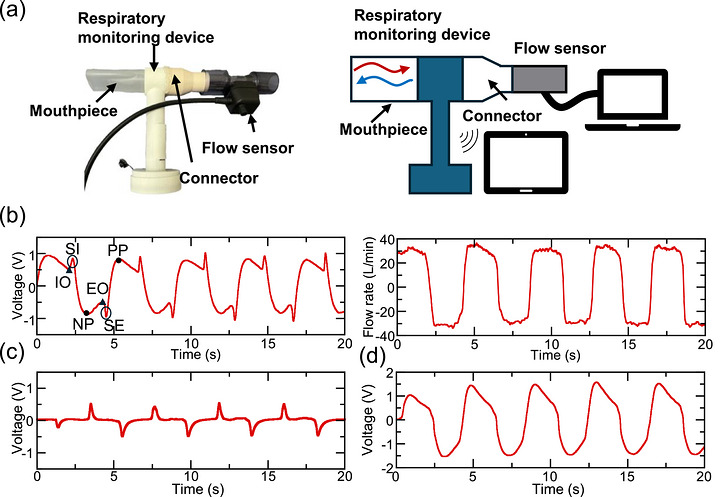
Experimental setup and representative results from healthy subjects (a,b), together with control experiment results for examining piezoelectric and pyroelectric contributions (c) and the effect of sensor orientation (d). (a) Photograph and schematic illustration of the experimental setup for oral breathing measurements. A mouthpiece is connected to the proposed device, and a commercially available flow sensor is connected downstream of the device for comparison. (b) Representative breathing waveform obtained from the proposed device (left) and the corresponding airflow rate signal recorded by the flow sensor (right) at an ambient temperature of 25.0°C. IO: inhalation onset, SI: spike of inhalation, NP: negative peak, EO: exhalation onset, SE: spike of exhalation, PP: positive peak. (c) Breathing waveform measured at an ambient temperature of 36.3°C. (d) Breathing waveform measured with the sensor reversed relative to the airflow direction (PZT layer facing downstream) at an ambient temperature of 25.0°C.

To understand the fundamental characteristics of the oral breathing signals acquired by the proposed device, measurements were conducted for 60 s on 15 healthy subjects with no history of respiratory disease and smoking in the past 10 years (12 males and 3 females, aged 21–63). For comparison, a commercially available flow sensor for adult oral respiratory monitoring was connected downstream of our device to simultaneously record airflow rate signals (Figure 3a).

Figure 3b shows a representative breathing waveform obtained from our device (left), together with the corresponding signal recorded simultaneously by the flow sensor (right). For clarity, only the initial 20 s segments of the 60 s recordings are shown to illustrate waveform features. In contrast to the flow sensor signal, the waveform acquired by our device exhibits spike‐like features, highlighted by circles, together with a broader peak within each breathing phase. Each inhalation onset (IO), as recognized by the subject at the start of inhalation, was consistently observed at a local minimum in the waveform, followed sequentially by a narrow positive spike (SI) and a broad negative peak (NP). Conversely, each exhalation onset (EO) occurred at a local maximum, followed by a narrow negative spike (SE) and a broad positive peak (PP).

#### 2.2.2 Origin of Spike Features

As described above, representative breathing waveforms include one spike and one peak with opposite polarities within each inhalation or exhalation phase. Based on signal polarities independently identified using purely mechanical or thermal stimuli (Figure 2), the piezoelectric response should be positive, and the pyroelectric response should be negative during inhalation, whereas the polarities should be reversed during exhalation. By directly comparing these polarity relationships with the measured breathing waveforms, the narrow spike and broad peak components can be attributed to the piezoelectric and pyroelectric responses, respectively.

To further validate this attribution, control experiments were performed to suppress the pyroelectric contribution by reducing the temperature difference between exhalation and inhalation, i.e., between body and ambient temperatures. Compared with the waveform measured at an ambient temperature of 25.0°C (Figure 3b, left), the broad peak components nearly disappeared at an ambient temperature of 36.3°C, close to body temperature, while the narrow spike components remained clearly observable (Figure 3c). This result directly validates the above attribution.

In addition, to confirm the influence of sensor orientation on the waveform characteristics, a comparative experiment was performed by reversing the sensor with respect to the airflow direction, i.e., with the PZT layer facing downstream. As shown in Figure 3d, the spike features observed in the normal configuration (Figure 3b, left) disappeared under the reversed orientation. This result is consistent with the expected polarity relationship between the piezoelectric and pyroelectric responses. Reversing the sensor orientation inverts the polarity of the piezoelectric response, causing it to align with that of the pyroelectric response and resulting in same‐polarity signal superposition without spike features. Hence, the breathing waveforms measured by the proposed device are characterized by sensor‐orientation‐dependent spike features, which originate from the piezoelectric response exhibiting polarities opposite to those of the simultaneously acquired pyroelectric response.

#### 2.2.3 Relationship Between Spike Features and Breathing Characteristics

The spike features observed shortly after inhalation or exhalation onset are expected to reflect rapid airflow changes during the initial transient phase of breathing. This is because the piezoelectric response, as read out by a charge amplifier, is sensitive to time‐varying deformation rather than static deformation [40, 41]. We therefore examined the relationship between spike features and the rate of airflow change during these transient phases. Because this rate cannot be directly controlled during voluntary breathing, it was indirectly modulated in a single representative subject under three conditions: i) natural breathing, ii) deeper breathing at approximately the same breathing frequency, and iii) faster breathing at approximately the same peak‐to‐valley amplitude. The breathing amplitude and frequency in the latter two conditions were adjusted relative to natural breathing and confirmed using simultaneously recorded flow sensor signals to remain within −6% of the intended constraint.

**FIGURE 4:**
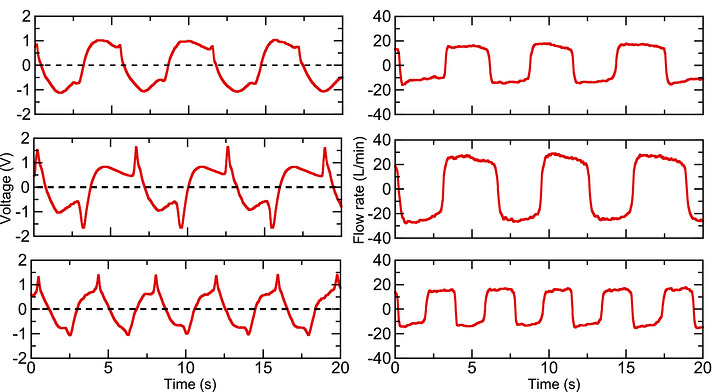
Control experiment results showing the correspondence between spike features and breathing under different depths and rates. Breathing waveforms measured by the proposed device (left) and the corresponding airflow rate signals measured by the flow sensor (right) for natural (upper), deeper (middle), and faster (lower) breathing.

Figure 4 shows the breathing waveforms measured by our device (left) and the corresponding airflow rate signals measured by the flow sensor (right). Compared with natural breathing (upper left), both deeper breathing (middle left) and faster breathing (lower left) resulted in a pronounced increase in spike amplitude. Quantitatively, the mean spike amplitude increased to approximately 2.1‐fold during deeper breathing and 1.2‐fold during faster breathing compared with natural breathing. Despite the different magnitudes, the observed increases in both conditions suggest that spike amplitude is more closely governed by the rate of airflow change immediately after inhalation or exhalation onset than by breathing depth or breathing rate alone.

### 2.3 Differences Among COPD, Asthma, and Healthy Subjects

We performed a pilot comparison of breathing waveforms acquired by our device among patients with COPD (3 males and 1 female, aged 63–83), patients with asthma (3 males and 2 females, aged 54–83), and the previously mentioned 15 healthy subjects. Subjects were included based on availability and informed consent, without waveform‐based preselection. Given the limited sample size, particularly in the patient groups, this analysis primarily focuses on characterizing natural breathing patterns in healthy individuals, using them as a reference to examine disease‐related deviations rather than to establish diagnostic criteria.

#### 2.3.1 Phase‐Resolved Metrics

**FIGURE 5:**
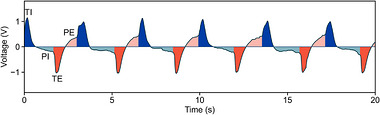
Representative breathing waveform from a COPD patient, showing characteristic differences from healthy subjects, together with the phase‐resolved segmentation used in the subsequent analysis. Each breathing cycle was segmented into four phases: transient inhalation (blue), post‐transient (quasi‐steady) inhalation (light blue), transient exhalation (red), and post‐transient (quasi‐steady) exhalation (light red). These phase‐resolved segments were used to compute the integral metrics R_TI_, R_PI_, R_TE_, and R_PE_.

A representative breathing waveform from patients over the initial 20 s is shown in Figure 5 to highlight characteristic differences from healthy subjects, while waveforms from the remaining patients are provided in Figure S2 (Supporting Information). Compared with healthy subjects (Figure 3b, left), this representative patient waveform shows an enhanced spike component, with increased amplitude and temporal contribution relative to the subsequent peak component. For clarity in the following analysis, the spike component is referred to as the transient phase, and the subsequent peak component as the post‐transient (quasi‐steady) phase.

To quantitatively characterize the relative contributions of the transient and post‐transient (quasi‐steady) phases, we defined phase‐resolved integral ratio metrics, R_TI_, R_PI_, R_TE_, and R_PE_, as illustrated in Figure 5. Specifically, each breathing cycle was segmented into four regions corresponding to the transient and quasi‐steady phases during inhalation and exhalation. The integrated area of each region was calculated and normalized by the total integrated area over the corresponding breathing cycle to obtain the metrics, which were then averaged over a 60 s recording period for each subject.

**FIGURE 6:**
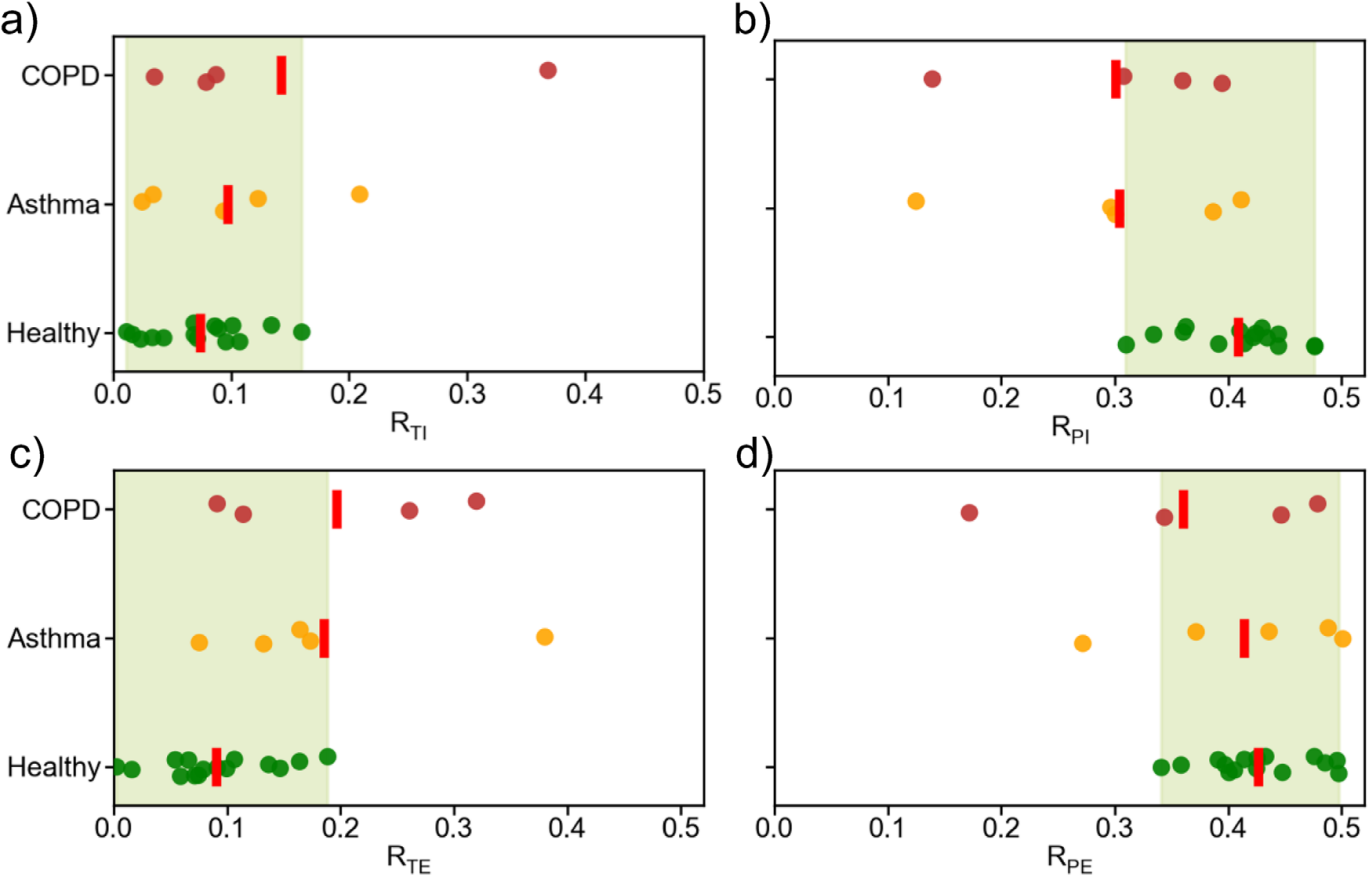
Jittered scatter plots of the phase‐resolved integral ratio metrics R_TI_ (a), R_PI_ (b), R_TE_ (c), and R_PE_ (d) for healthy subjects and patients with COPD or asthma.

Figure 6 shows jittered scatter plots of the phase‐resolved integral ratio metrics for healthy subjects and patients with COPD or asthma. Healthy subjects exhibited a narrow and well‐defined distribution across all four metrics, as highlighted by the shaded reference region. Specifically, the transient‐phase ratios (R_TI_ and R_TE_) remained low, typically within 0–20%, whereas the quasi‐steady‐phase ratios (R_PI_ and R_PE_) accounted for a larger fraction of the breathing cycle, typically within 30–50%. In contrast, although some patients exhibited values comparable to those of healthy subjects, a subset displayed pronounced deviations beyond the healthy reference region characterized by increased transient‐phase ratios (approximately 20–40%) and reduced quasi‐steady‐phase ratios (approximately 10–30%). Such deviations were observed slightly more frequently in the inhalation quasi‐steady phase (R_PI_) and the exhalation transient phase (R_TE_) than in the inhalation transient phase (R_TI_) and the exhalation quasi‐steady phase (R_PE_). This tendency was also reflected in the group‐averaged values, with more pronounced differences between patients and healthy subjects for R_PI_ and R_TE_ than for R_TI_ and R_PE_.

Overall, the proposed phase‐resolved integral ratio metrics indicate that healthy subjects exhibit a breathing pattern dominated by quasi‐steady phases, whereas patients tend to show a relative shift toward transient‐enhanced breathing. This shift is slightly more pronounced as a reduced quasi‐steady contribution during inhalation and an enhanced transient contribution during exhalation.

#### 2.3.2 Conventional Breathing Descriptors

To provide a baseline comparison with the proposed phase‐resolved metrics, we analyzed a set of conventional breathing descriptors commonly used in portable or wearable breathing monitoring. These included time‐domain breathing metrics (inhalation, exhalation, and breathing cycle durations), amplitude‐related metrics (peak, valley, and peak‐to‐valley amplitudes), and their cycle‐to‐cycle variability quantified by the coefficient of variation (CV), defined as the ratio of the standard deviation to the mean. In addition, waveform asymmetry was characterized using skewness, and frequency‐domain characteristics were evaluated using the relative contribution of high‐frequency components.

**FIGURE 7:**
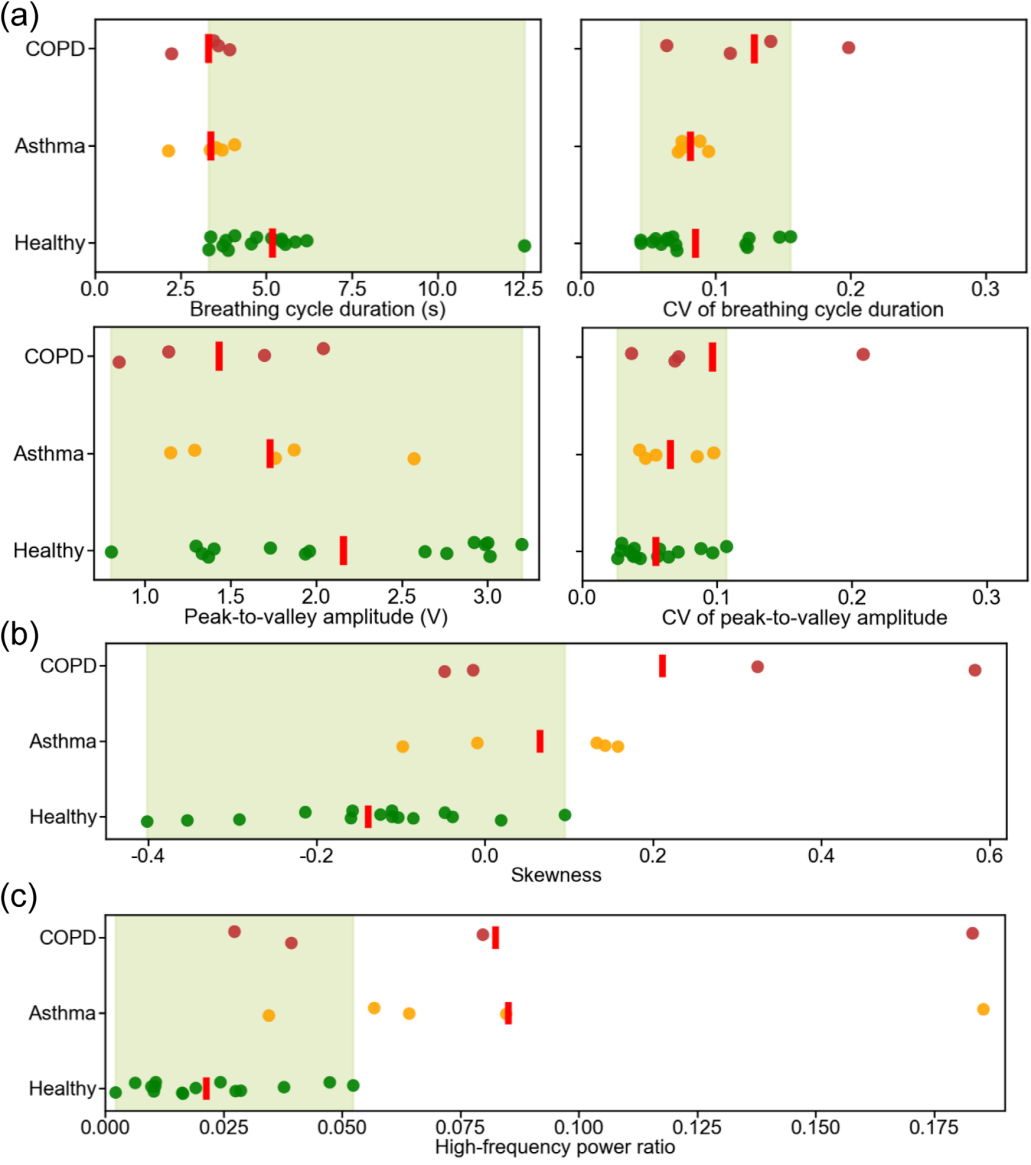
Jittered scatter plots of conventional breathing descriptors for healthy subjects and patients with COPD or asthma. (a) Breathing cycle duration and peak‐to‐valley amplitude, together with their corresponding cycle‐to‐cycle coefficients of variation (CV). (b) Skewness of the breathing waveforms. (c) Relative contribution of high‐frequency components.

Figure 7a presents jittered scatter plots of breathing cycle duration and peak‐to‐valley amplitude, together with their corresponding CVs. Other time‐domain and amplitude‐related metrics obtained from separated inhalation and exhalation phases showed consistent trends and are therefore provided in Figure S3 (Supporting Information). In contrast to the phase‐resolved metrics, both breathing duration and amplitude exhibited a broad distribution among healthy participants, indicating substantial inter‐individual variability under natural breathing conditions. For the patient groups, breathing cycle duration values were narrowly distributed near the lower bound of the healthy reference range, with one COPD and one asthma subject falling below this range. In comparison, peak‐to‐valley amplitude values for all patients showed a dispersed distribution but remained fully within the healthy reference range. At the group level, the average amplitude exhibited a gradual decrease from healthy subjects to asthma and further to COPD subjects. Compared with the duration and amplitude, the corresponding CVs displayed a relatively narrow distribution among healthy subjects, defining a stable reference range. Most patients fell within this healthy reference range, with only one COPD subject exceeding the healthy range for the CVs of both duration and amplitude. These results suggest that, on average, patients tend to show faster, shallower, and more variable breathing patterns compared with healthy subjects.

Figure 7b,c show jittered scatter plots of skewness and the relative contribution of high‐frequency components. FFT amplitude spectra of patients outside the healthy reference range for high‐frequency power ratio are provided in Figure S4 (Supporting Information). Skewness values of healthy subjects were distributed over a relatively wide range, with the majority in the negative range, whereas those of patient groups tended to shift toward positive values, with two COPD subjects markedly exceeding both the healthy and asthma ranges. Inspection of the waveforms from the out‐of‐range subjects revealed that the positive skewness arises from relatively larger quasi‐steady exhalation peaks compared with the corresponding inhalation peaks. For the relative contribution of high‐frequency components, healthy subjects typically exhibited values below 5%, whereas all COPD subjects and two of the five asthma subjects exceeded this range, with values reaching up to approximately 19%. This indicates the presence of more pronounced rapid intra‐cycle fluctuations within individual breathing waveforms in a subset of patients.

#### 2.3.3 Quantitative Analysis and Physiological Interpretation

##### 2.3.3.1 Quantitative Comparison of Breathing Features

**FIGURE 8:**
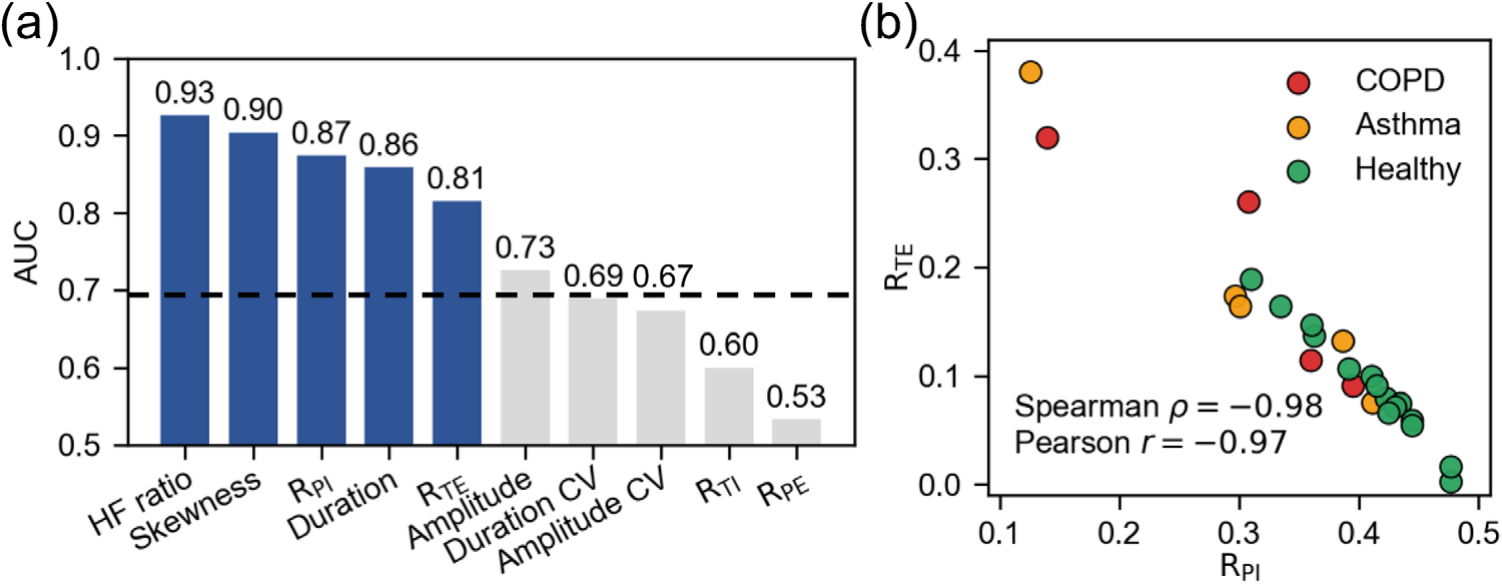
(a) Area under the receiver operating characteristic curve (AUC) for distinguishing the healthy group (*n* = 15) from the patient group with COPD and asthma (*n* = 9). HF ratio: high‐frequency power ratio; Duration: breathing cycle duration; Amplitude: peak‐to‐valley amplitude. The dashed line at AUC = 0.70 is shown as a reference level for moderate discriminative performance. (b) Relationship between R_PI_ and R_TE_ across all subjects.

To quantitatively evaluate the discriminative performance of the extracted breathing features, we calculated the area under the receiver operating characteristic curve (AUC), along with Cliff's *δ* as a complementary measure of effect size (Table S2). Given the limited sample sizes of the COPD and asthma groups, these two groups were combined into a single patient group (*n* = 9) for exploratory comparison with the healthy group (*n* = 15). Figure 8a summarizes the AUC values of the extracted features. Several features showed relatively high group‐level discriminative performance, including the high‐frequency power ratio, skewness, R_PI_, breathing cycle duration, and R_TE_. The relatively high AUC of breathing cycle duration is consistent with the group‐level trend toward shorter breathing cycles in patients, despite the overlap between groups observed in Figure 7a. Among the phase‐resolved metrics, R_PI_ and R_TE_ showed higher AUC values than R_TI_ and R_PE_, consistent with the group‐level differences observed in Figure 6.

##### 2.3.3.2 Coupling Between Quasi‐Steady Inhalation and Transient Exhalation

We further examined the relationship between R_PI_ and R_TE_, the two phase‐resolved metrics with relatively high AUC values. As shown in Figure 8b, a strong inverse coupling was observed across all subjects (Spearman's *ρ* = −0.98; Pearson's *r* = −0.97). Because the four phase‐resolved metrics are normalized components whose sum equals one, inverse relationships among them are partly expected. However, the R_PI_–R_TE_ correlation was stronger than the R_PI_–R_TI_ and R_PI_–R_PE_ correlations (*ρ* = −0.42; *r* = −0.60 and *ρ* = 0.36; *r* = 0.55, respectively). This suggests that, in this pilot dataset, the phase redistribution in the patient group was mainly characterized by a coupled decrease in quasi‐steady inhalation contribution and an increase in transient exhalation contribution.

##### 2.3.3.3 Physiological Interpretation

The observed differences across phase‐resolved integral metrics and conventional descriptors can be interpreted in the context of known respiratory physiology, which helps clarify their respective information content and inherent limitations.

Under natural breathing conditions, breathing rate, breathing depth, and their cycle‐to‐cycle variability are primarily influenced by individual breathing habits and momentary physiological conditions. Accordingly, substantial inter‐individual variability is observed even among healthy subjects. Patients showed a group‐level tendency toward faster, shallower, and more variable breathing, and the AUC analysis suggests that breathing cycle duration provides useful group‐level discriminative information. However, the considerable overlap with healthy subjects indicates that timing‐, amplitude‐, and CV‐based descriptors alone remain limited for reliable discrimination at the individual level. Moreover, these descriptors mainly describe aggregate breathing characteristics.

By comparison, skewness and high‐frequency content primarily reflect disturbances in intra‐cycle breathing dynamics that arise from the internal structure of the breathing waveform. While shaped by breathing behavior, these descriptors are less amenable to intentional modulation and instead reflect altered inspiratory–expiratory phase distribution and intra‐cycle stability. The larger deviations observed in COPD than in asthma are physiologically plausible, given the chronic and largely irreversible nature of airflow limitation in COPD compared with the typically reversible and episodic obstruction in asthma. However, these descriptors summarize overall waveform morphology without explicitly resolving the contributions of individual breathing phases.

The phase‐resolved integral metrics further extend this interpretation by explicitly resolving how breathing effort is distributed between transient and quasi‐steady phases during inspiration and expiration. The ability to separate these phases is enabled by the characteristic waveform captured by the proposed device, which contains distinct transient and quasi‐steady components within each breathing direction. The relatively high discriminative performance of R_PI_ and R_TE_, together with their strong inverse coupling, suggests that patient‐associated waveform changes involve a phase‐specific redistribution of breathing dynamics. Importantly, skewness and high‐frequency content can be viewed as indirect manifestations of this redistribution, whereas the phase‐resolved metrics provide a more direct and interpretable representation of it.

Taken together, these results suggest a hierarchy of respiratory information: duration, amplitude, and their variability primarily reflect aggregate and behavior‐sensitive breathing characteristics; skewness and high‐frequency content capture altered intra‐cycle dynamics; and phase‐resolved integral metrics directly characterize the underlying redistribution of breathing phases. This conceptual progression highlights the added value of phase‐resolved analysis for probing respiratory dynamics that are less accessible through conventional descriptors alone.

## 3 Conclusion

This work presents a portable breathing monitoring device based on a flexible PZT sensor that simultaneously captures pressure‐ and temperature‐related airflow responses during natural oral breathing. By exploiting polarity‐opposed piezoelectric and pyroelectric responses through sensor orientation, the recorded breathing waveforms exhibit a characteristic dual‐component structure, comprising a narrow transient spike and a broad quasi‐steady peak with opposite polarities during inhalation and exhalation. This intrinsic waveform structure enables direct access to how breathing dynamics are distributed within each breathing cycle, beyond aggregate measures such as breathing rate or depth. On this basis, phase‐resolved integral metrics were introduced to quantify the relative contribution of transient and quasi‐steady components during inhalation and exhalation. Pilot comparisons among healthy subjects and patients with COPD or asthma, together with exploratory AUC and correlation analyses, suggest that the proposed phase‐resolved metrics capture patient‐associated waveform changes as a phase‐specific redistribution of breathing dynamics, particularly a coupled decrease in quasi‐steady inhalation contribution and increase in transient exhalation contribution. More broadly, the proposed dual‐response sensing approach shifts natural breathing monitoring from aggregate, behavior‐sensitive features toward intra‐cycle airflow dynamics that are more directly linked to airway mechanics and respiratory system function.

Although the present study is limited in scale and not intended to establish diagnostic criteria, it demonstrates the potential of dual‐response, phase‐resolved respiratory sensing to access functional changes in natural breathing that are not readily accessible using conventional flow‐based monitoring. Future validation in larger and more diverse populations will be essential to establish the robustness and physiological relevance of the proposed approach and to support its translation toward practical respiratory monitoring in daily‐life settings.

## 4 Materials and Methods

### 4.1 Sensor Materials and Fabrication

As shown in Figure 1a, the sensor element was encapsulated between two polyimide films for protection. The sensor element consisted of a 20 µm thick fluorophlogopite mica [KMg_3_(AlSi_3_O_10_)F_2_] substrate, a 2 µm thick PbZr_0.52_Ti_0.48_O_3_ (PZT) film, and parallel‐plate platinum electrodes (100 nm thick) above and below the PZT layer. The electrodes were fabricated by magnetron sputtering, and the PZT film was formed using a transfer‐free sol‐gel method [42]. The detailed fabrication process followed that reported in our previous work [38].

### 4.2 Signal Conditioning and Data Acquisition

The electrical charges generated by the sensor were converted into voltage signals using an onboard charge amplifier with a 1.65 V bias applied, resulting in an output voltage range of 0–3.3 V. In subsequent signal processing, this bias offset was subtracted, and the voltage waveforms were treated as effective bipolar signals in the range of ±1.65 V. The amplified signals were digitized by an onboard 12‐bit analog‐to‐digital converter and transmitted wirelessly via Bluetooth to an iOS‐based mobile terminal for recording. The sampling interval was 3.2 ms. All circuit configurations and acquisition parameters followed those reported in our previous work [38].

### 4.3 Setup and Protocols of Breathing Measurements

Breathing measurements were performed in both healthy volunteers and patients with diagnosed respiratory diseases using a unified experimental protocol. To accommodate the slightly larger outer diameter (26 mm) of the mouthpieces used in the hospital, the inner diameter of the flow channel in the proposed PZT‐based device used for patient measurements was slightly increased compared with that used for healthy subjects, while all other components and acquisition settings were identical. The characteristic waveform features were consistently observed across both device form factors. For measurements in healthy volunteers conducted in a laboratory setting, a commercially available oral respiratory flow sensor (SFM3300‐AW, Sensirion, Figure 3a) was used to simultaneously record airflow rate signals (100 Hz) on a personal computer. Due to practical constraints in the clinical environment, simultaneous flow sensor measurements were not performed during patient measurements.

All measurements, except for specifically noted control experiments, were conducted under seated, resting conditions in an indoor environment at room temperature. Participants were instructed to breathe naturally through a mouthpiece connected to the proposed device. To minimize voluntary modulation of breathing, visual feedback of the recorded signals was not provided during the measurements. Each recording lasted approximately 60 s, which was sufficient to capture multiple consecutive breathing cycles under stable conditions. For healthy volunteers, the use of a nasal clip was determined on an individual basis depending on their ability to maintain natural oral breathing; for patient measurements, a nasal clip was consistently used.

All breathing measurements were conducted in accordance with the Declaration of Helsinki and were approved by the Graduate School of Informatics, Nagoya University (approval number: hc24‐12), as well as by the institutional review board of the participating hospital. Written informed consent was obtained from all participants for participation in the study and publication of anonymized data. No patient‐identifiable information is included in this article.

### 4.4 Phase Segmentation of Breathing Cycles

Each breathing cycle was segmented into four phase‐resolved regions used to compute R_TI_, R_PI_, R_TE_, and R_PE_. Segmentation was based on characteristic features of the voltage waveform. The onset of inhalation or exhalation was first identified, appearing as a local minimum or maximum, respectively, reflecting signal polarity reversal at the phase transition. When clear local extrema were absent, the onset was identified as an inflection point characterized by a pronounced change in signal slope accompanying polarity reversal. If multiple local extrema were present, the one immediately preceding the spike feature was selected, as the spike consistently occurred shortly after the phase transition. Owing to subject‐dependent waveform variations and occasional local noise, inhalation and exhalation onsets were determined by visual inspection to ensure consistent and physiologically reasonable phase assignment across recordings. The transient phase was defined as the interval from the inhalation or exhalation onset to the subsequent zero‐crossing of the voltage signal (0 V). The remaining interval until the onset of the next inhalation or exhalation phase was defined as the quasi‐steady phase. Although this rule‐guided manual segmentation was sufficient for the present pilot analysis, development of an automated segmentation algorithm will be necessary for large‐scale studies and practical deployment.

### 4.5 Frequency‐Domain Analysis

The relative contribution of high‐frequency components was calculated by Fourier analysis of the breathing waveforms. Prior to transformation, a Hann window was applied to reduce spectral leakage. The frequency range was set to 0.05–2.0 Hz, divided into a low‐frequency band (0.05–0.6 Hz) and a high‐frequency band (0.6–2.0 Hz). The boundary frequency of 0.6 Hz was chosen to remain above the fundamental breathing frequency even under fast breathing conditions, while avoiding contamination from higher‐order harmonics.

## Conflicts of Interest

The authors declare no conflict of interest.

## Supporting information




**Supporting File**: adhm71282‐sup‐0001‐SuppMat.docx.

**Supporting Movie**: adhm71282‐sup‐0002‐MovieS1.mp4.

## Data Availability

The data that support the findings of this study are available on request from the corresponding author upon reasonable request.
