# Supplementary material for: Portable Breathing Monitoring With Phase‐Resolved Airflow Dynamics Enabled by a Dual‐Response Flexible PZT Sensor
Source: Adv Healthc Mater. 2026 May 28;15(25):e71282. doi: 10.1002/adhm.71282 (PMC13331601; doi:10.1002/adhm.71282)
Supplement: Supplementary file 1 — Supporting File: adhm71282‐sup‐0001‐SuppMat.docx. [file ADHM-15-0-s002.docx]

Supporting Information

**Portable Breathing Monitoring with Phase-Resolved Airflow Dynamics Enabled by a Dual-Response Flexible PZT Sensor**

Minyu Li, Jun Aoyama, Yuchao Wu, Tomomi Uchiyama, Kosho Yoshikawa, Toshiki Mano, Yuxi Song, and Hedong Zhang*

M. Li, J. Aoyama, Y. Wu, Y. Song, H. Zhang

Department of Complex Systems Science, Graduate School of Informatics, Nagoya University

Furo-cho, Chikusa-ku, Nagoya 464-8601, Japan

E-mail: zhang@i.nagoya-u.ac.jp

T. Uchiyama

Institute of Materials and Systems for Sustainability, Nagoya University

Furo-cho, Chikusa-ku, Nagoya 464-8601, Japan

K. Yoshikawa

Department of Respiratory Medicine, Daido Hospital, Kojunkai Social Medical Corporation

9 Hakusui-cho, Minami-ku, Nagoya 457-8511, Japan

T. Mano

Chuo Graduate School of Strategic Management, Chuo University

742-1 Higashinakano, Hachioji-shi, Tokyo 192-0393 Japan

**Note S1. Comparison of Pyroelectric Sensitivity between Flexible PZT and PVDF Sensors**

The pyroelectric sensitivity of the flexible PZT was compared with that of a commercially available polyvinylidene fluoride (PVDF) sensor. A cyclic temperature variation of the sensor surface between 25.0 and 36.4 °C over 2 s was applied using the same thermal excitation method as illustrated in Figure 2b of the main text. Except for replacing the sensor, all other conditions were kept identical. The average peak-to-valley pyroelectric amplitude was 0.37 ± 0.039 V for the PZT sensor and 0.59 ± 0.081 V for the PVDF sensor, indicating comparable pyroelectric sensitivity (**Figure S1**).

**Figure S1.** Pyroelectric voltage responses of flexible PZT (a) and PVDF (b) sensors measured under identical thermal excitation. A photograph of the PVDF sensor is shown in (b).

**Note S2. Neutral Plane Calculation of the Flexible PZT Sensor**

The position of the neutral mechanical plane of the flexible PZT sensor was calculated based on its multilayer structure (Figure 1a in the main text and **Table S1**) using the following equation:

$$\begin{aligned} y_{N}=\frac{\sum_{i=1}^{n} E_{i}b_{i}t_{i}y_{i}}{\sum_{i=1}^{n} E_{i}{b_{i}t}_{i}}\#\left( S1 \right) \end{aligned}$$

where *E_i_*, *b_i_*, *t_i_* are the Young’s modulus, width, and thickness of each layer, respectively, and *y_i_* is the distance from the center plane of each layer to the top surface of the first layer. Using the values listed in Table S1, the neutral plane was calculated to be approximately 15.1 μm from the top surface of the first layer, lying within the mica layer.

**Table S1** Material, thickness, width, and Young’s modulus of each layer of the flexible PZT sensor. The Young’s modulus values were taken from Refs. [1–4].

| Layer No. | Material | Thickness (μm) | Width (mm) | Young’s modulus (GPa) |
| --- | --- | --- | --- | --- |
| 1 | Polyimide film | 10.0 | 15.0 | 2.5 |
| 2 | Pt | 0.1 | 10.0 | 116 |
| 3 | PZT | 2.0 | 10.0 | 140 |
| 4 | Pt | 0.1 | 10.0 | 116 |
| 5 | Mica | 20.0 | 10.0 | 5.4 |
| 6 | Polyimide film | 10.0 | 15.0 | 2.5 |

**Figure S2.** Breathing waveforms from eight patients measured by the proposed device. The waveform of the remaining patient is shown in Figure 5 of the main text.

**Figure S3.** Jittered scatter plots of inhalation duration, exhalation duration, peak amplitude, valley amplitude, together with their corresponding cycle-to-cycle coefficients of variation (CV), for healthy subjects and patients with COPD or asthma.

**Figure S4.** FFT amplitude spectra of patients whose high-frequency power ratios fall outside the healthy reference range, as defined in Figure 7c of the main text.

**Table S2** Cliff’s *δ* and area under the receiver operating characteristic curve (AUC) values of extracted breathing features for distinguishing the healthy and patient groups. Features are listed in descending order of AUC. Positive and negative Cliff’s *δ* values indicate higher feature values in the patient and healthy groups, respectively.

| Feature | Cliff’s *δ* | AUC |
| --- | --- | --- |
| High-frequency power ratio | 0.85 | 0.93 |
| Skewness | 0.81 | 0.90 |
| R_PI_ | −0.75 | 0.87 |
| Breathing cycle duration | −0.72 | 0.86 |
| R_TE_ | 0.63 | 0.81 |
| Peak-to-valley amplitude | −0.45 | 0.73 |
| Breathing cycle duration CV | 0.38 | 0.69 |
| Peak-to-valley amplitude CV | 0.35 | 0.67 |
| R_TI_ | 0.20 | 0.60 |
| R_PE_ | −0.07 | 0.53 |

**References**

1. Yang, S.-Y., “Mechanical Properties of Polyimide Films,” in *Advanced Polyimide Materials: Synthesis, Characterization, and Applications*, ed. S.-Y. Yang (Oxford: Elsevier, 2018).
2. Abbas, K., Alaie, S., Ghasemi Baboly, M., Elahi, M. M. M., Anjum, D. H., Chaieb, S., and Leseman, Z. C., “Nanoscale Size Effects on the Mechanical Properties of Platinum Thin Films and Cross-Sectional Grain Morphology,” *Journal of Micromechanics and Microengineering* 26, no. 1 (2016): 015007. <https://doi.org/10.1088/0960-1317/26/1/015007>
3. Casset, F., *et al*., “Young’s Modulus and Poisson Ratio of PZT Thin Film by Picosecond Ultrasonics,” in *Proceedings of the 2012 IEEE International Ultrasonics Symposium*, Dresden, Germany (2012): 2180–2183. <https://doi.org/10.1109/ULTSYM.2012.0544>
4. Jin, D. W., Ko, Y. J., Kong, D. S., Kim, H. K., Ha, J.-H., Lee, M., Hong, J.-I., and Jung, J. H., “Thermal Stability and Young’s Modulus of Mechanically Exfoliated Flexible Mica,” *Current Applied Physics* 18, no. 12 (2018): 1486–1491. <https://doi.org/10.1016/j.cap.2018.09.002>
